# Acknowledgement of Editorial board members

**DOI:** 10.14252/foodsafetyfscj.D-24-00016

**Published:** 2024-12-20

The Editor-in-chief of ***Food Safety*** would like to thank all our Editorial board members who have contributed to the journal in 2024.

**Table:** 

AKAIKE Akinori	KASAI Midori	OGAWA Kumiko	UMEMURA Takashi
AKIYAMA Hiroshi	KARITA Kanae	ONO Atsushi	WANIBUCHI Hideki
AOKI Yasunobu	KIMURA Bon	OSAKA Ken	YAMANAKA Noriko
AOYAMA Hiroaki	KOSEKI Shigenobu	OZAKI Hiroshi	YAMATE Jyoji
ARAKAWA Yoshichika	KUMAGAI Yoshito	OZEKI Yoshihiro	YOGO Yasuhiro
ASAI Tetsuo	KUSHIRO Masayo	SAITO Yoshiro	YOKOI Tsuyoshi
ENDO Ginji	KOJIMA Tokiko	SHIBUTANI Makoto	YOKOYAMA Takashi
GODA Yukihiro	MARUYAMA Aya	SHIMIZU Makoto	YOSHIDA Midori
HARA-KUDO Yukiko	MISAWA Naoaki	SUGIMOTO Naoki	Amnart Poapolathep
HIRATSUKA Akira	MITSUMORI Kunitoshi	SUGITA-KONISHI Yoshiko	Frederick A. Beland
HIROSE Akihiko	MIZUSAWA Hidehiro	SUZUKI Toshiyuki	George E.N. Kass
HONMA Masamitsu	NAKAJIMA Harushi	TERAJIMA Jun	Keerthi Siri Guruge
HORIMOTO Masao	NISHIURA Hiroshi	TESHIMA Reiko	Weida Tong
IMAI Toshio	NOHARA Keiko	TODA Miou	Yun Yun Gong
ISHII Rie	NOHMI Takehiko	UEMA Masashi	

